# Supplementary figures and images for: Identification of a Functional Non-coding Variant in the GABAA Receptor α2 Subunit of the C57BL/6J Mouse Reference Genome: Major Implications for Neuroscience Research
Source: Front Genet. 2019 Mar 29;10:188. doi: 10.3389/fgene.2019.00188 (PMC6449455; doi:10.3389/fgene.2019.00188)

A.

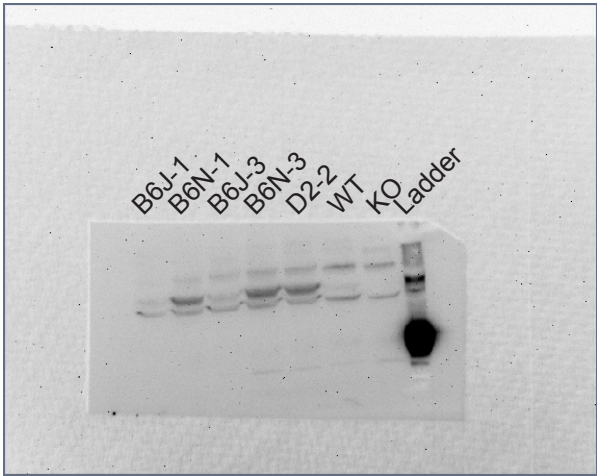

B.

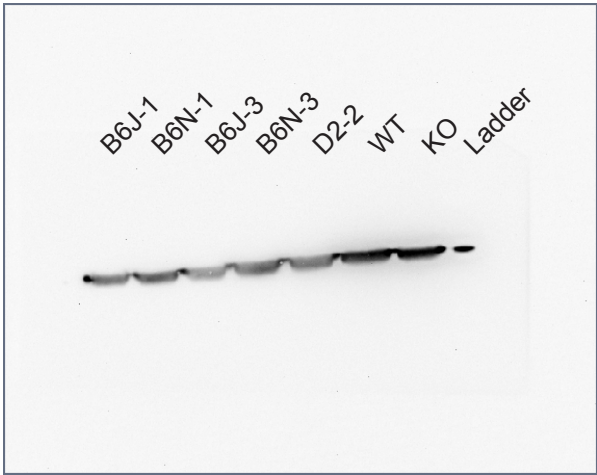

C.

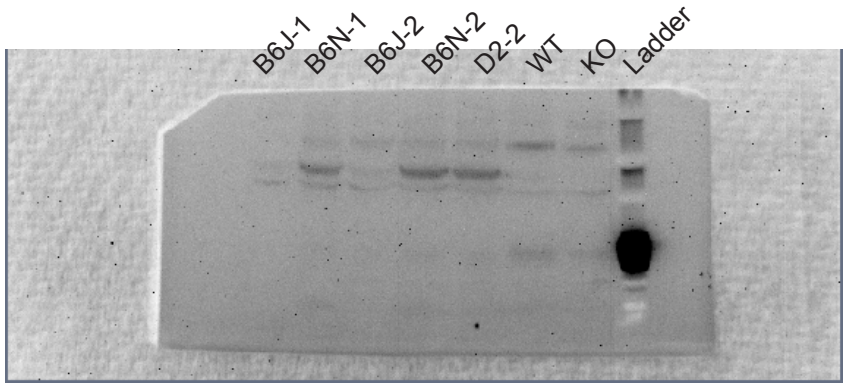

D.

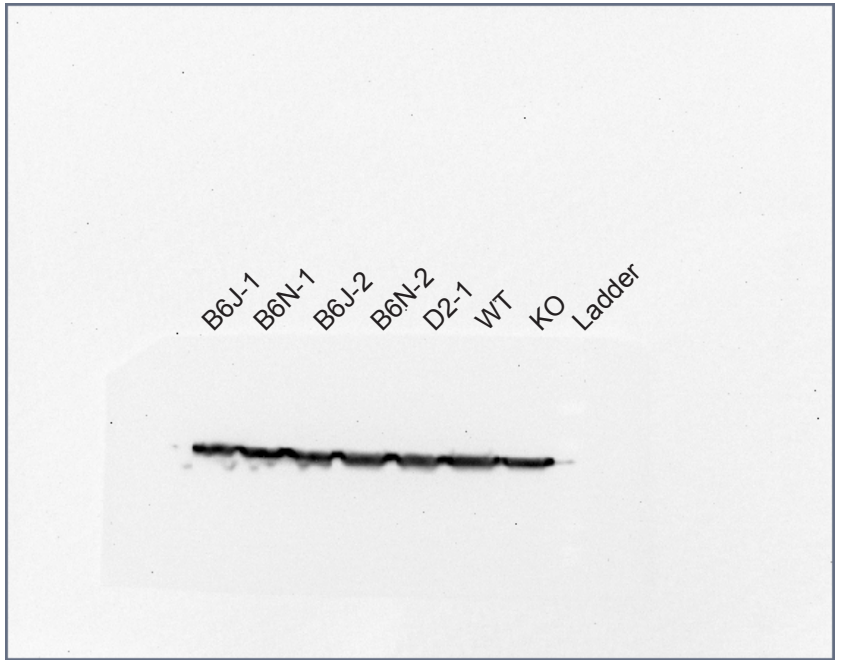

Supplement: FIGURE S1 — Original blots from Figure 1H analysis. Blots were incubated overnight with anti-GABRA2 antibody (PhosphoSolutions #822-GA2CL), followed by a horseradish peroxidase-conjugated anti-rabbit secondary, and developed on a BioRad ChemiChem chemiluminescent detection system. (A) Unedited whole-blot GABRA2 staining. Following development, blots were stripped and re-probed for GAPDH (Fitzgerald #10R-G109A) as a loading control, and developed using the same protocol. (B) Unedited whole-blot GAPDH staining. (C,D) show equivalent whole-blot unedited images from a second replicate of the experiment. Samples B6J-1 and B6N-1 were run during both experiments. [file Data_Sheet_1.PDF]

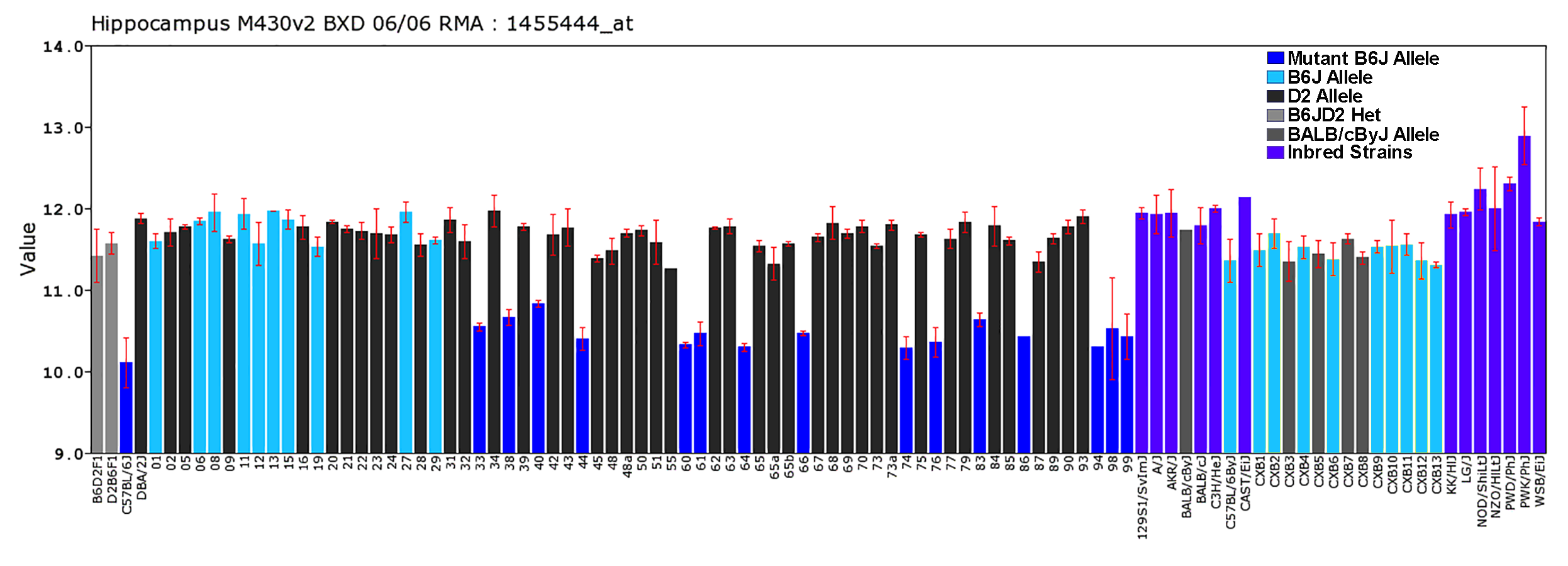

Supplement: FIGURE S3 — Strain distribution pattern of expression of Gabra2. A 2 to 3-fold reduction in expression of mRNA levels in hippocampus [GN110 Hippocampus Consortium M430v2 (Jun06) RMA] that is only segregating in the new BXD strains (BXD33 and higher) that have inherited the Gabra2 B6J private mutation. The Y-axis provides an estimate of the expression of Gabra2 (Affymetrix probe set 1455444_at) on log2 scale. To the far left are the two reciprocal F1 hybrids between B6J and D2 with comparatively normal high expression, demonstrating a dominant phenotype of the wild type D allele. Note that all of the initial set of BXDs (from BXD1 to BXD32) have high expression, a finding only compatible with the hypothesis that the mutation in B6J occurred after the inception of these strains. In contrast, 15 of the newer BXD strains have low expression. Finally, all other common inbred strains, including both B6ByJ and all parental strains of the Collaborative Cross other than B6J have high expression. High expression is also evident in the CXB recombinant inbred panel generated by crossing BALB/cByJ and B6ByJ. Bars colored by genotype or inbred strain. BXD genotype determined using marker rs13478320 located on Chr 5 at 70,742,059 bp. CXB genotype determined using marker rs13478300 located on Chr 5 at 65,043,224 bp. [file Image_3.tif]

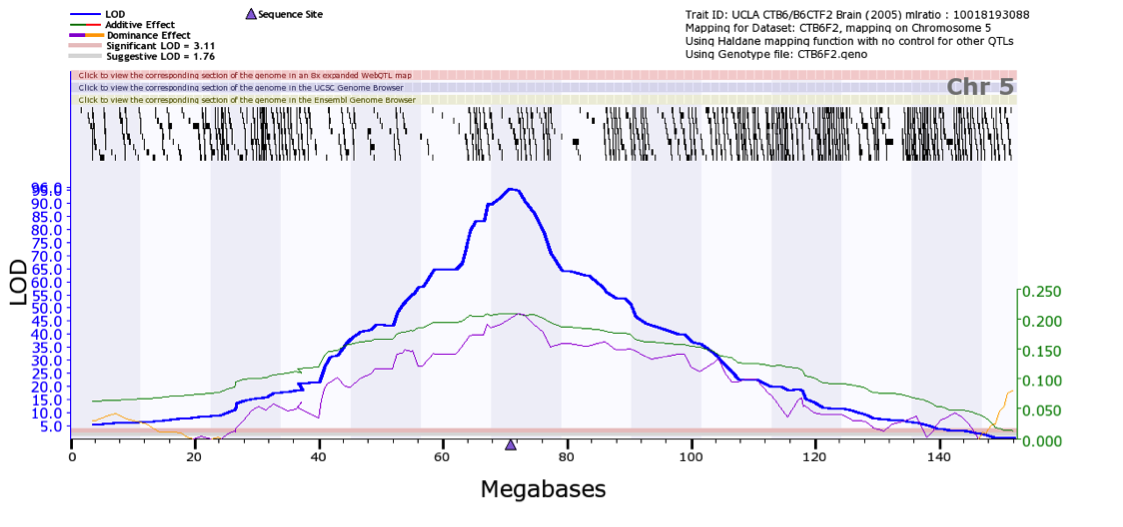

Supplement: FIGURE S4 — Mode of inheritance of Gabra2 alleles. In a large eQTL transcriptome analysis of 400 F2 intercross progeny between B6J and CAST/EiJ, the LOD peak is precisely aligned on the Gabra2 gene (purple triangle), the effect size is about 0.20 z per allele (right Y-axis). Note also that the dominance effect is complete (compare peak of the purple and green effect size plots). [file Image_4.png]
