# Supplementary material for: Identification of a Functional Non-coding Variant in the GABAA Receptor α2 Subunit of the C57BL/6J Mouse Reference Genome: Major Implications for Neuroscience Research
Source: Front Genet. 2019 Mar 29;10:188. doi: 10.3389/fgene.2019.00188 (PMC6449455; doi:10.3389/fgene.2019.00188)
Supplement: FIGURE S2 — Original blots from founder mouse analysis in Figure 3B. Blots were incubated overnight with anti-GABRA2 (PhosphoSolutions #822-GA2CL) and anti-GAPDH (Fitzgerald #10R-G109A) antibodies, followed by fluorescent-conjugated secondary antibodies, and developed on an Odyssey imaging system. (A,B) show unedited whole-blot GABRA2 and GAPDH staining of cortex samples, respectively. (C,D) show corresponding GABRA2 and GAPDH uncropped whole-blot images that have been edited using Photoshop’s brightness/contrast and levels features to make bands visible. (E–H) show equivalent images for blots containing hippocampal samples. [file Data_Sheet_2.PDF]

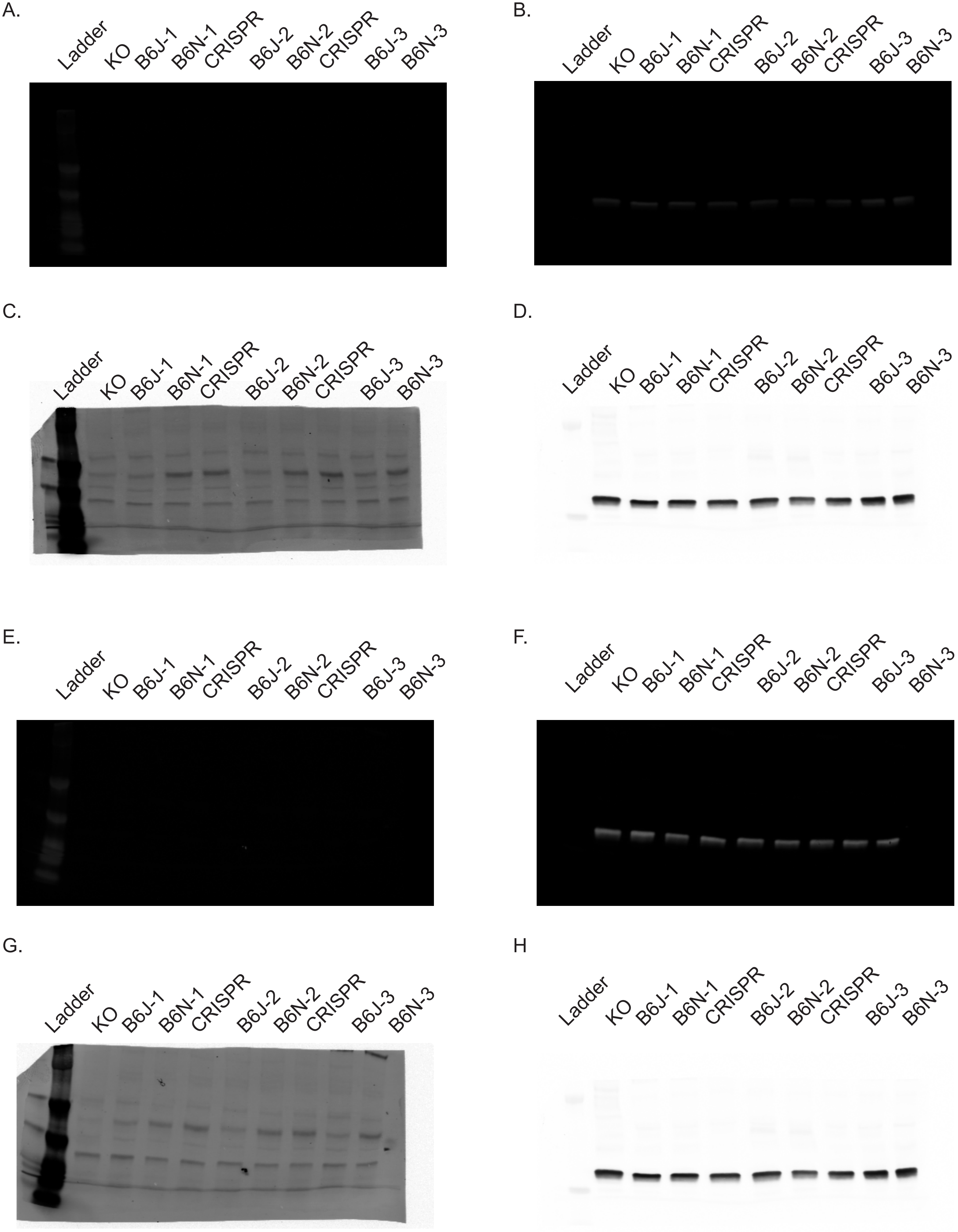
A.
Ladder
KO
B6J-1
B6N-1
CRISPR
B6J-2
B6N-2
CRISPR
B6J-3
B6N-3
B.
Ladder
KO
B6J-1
B6N-1
CRISPR
B6J-2
B6N-2
CRISPR
B6J-3
B6N-3
C.
Ladder
KO
B6J-1
B6N-1
CRISPR
B6J-2
B6N-2
CRISPR
B6J-3
B6N-3
D.
Ladder
KO
B6J-1
B6N-1
CRISPR
B6J-2
B6N-2
CRISPR
B6J-3
B6N-3
E.
Ladder
KO
B6J-1
B6N-1
CRISPR
B6J-2
B6N-2
CRISPR
B6J-3
B6N-3
F.
Ladder
KO
B6J-1
B6N-1
CRISPR
B6J-2
B6N-2
CRISPR
B6J-3
B6N-3
G.
Ladder
KO
B6J-1
B6N-1
CRISPR
B6J-2
B6N-2
CRISPR
B6J-3
B6N-3
H
Ladder
KO
B6J-1
B6N-1
CRISPR
B6J-2
B6N-2
CRISPR
B6J-3
B6N-3
